# Automated Imaging Screen Reveals Promising Drug Candidates

**DOI:** 10.1371/journal.pbio.0030165

**Published:** 2005-04-05

The birth of combinatorial chemistry in the early 1990s held out the promise that scientists would soon synthesize trillions of compounds at a time and screen up to a million a day, revolutionizing the process of drug discovery. But synthesizing a vast library of compounds is just the first step in the historically painstaking process of determining whether a compound has the desired effect on a target. In addition to an ever-growing library of candidate therapeutic compounds, advances in genome analysis have produced a growing list of potential drug targets—drowning drug researchers in an excess of riches.

In a new study, Kevan Shokat and colleagues report a high-throughput screening method that substantially narrows the field of candidate therapeutic agents. Their approach takes advantage of a recently developed automated system (called Cytometrix) that combines advanced imaging and bioinformatics approaches to classify cells according to small-molecule-induced changes in cell size, shape, and structure (morphology). Their analysis identified a novel compound with promising potential as an anticancer agent.

The Cytometrix system offers a high-throughput, unbiased (that is, machine-rendered) approach to identifying molecules that induce changes in cell processes, molecules that could be used to probe cells or to test for therapeutic effect. High-tech imaging equipment, combined with statistical analysis, extracts the biological effects of small molecules as “phenotypic readouts” based on the physical and structural characteristics of the cells. Using this system, the authors tested 107 small-molecule compounds with structural similarities to four types of protein kinase inhibitors—used in anticancer therapies—by injecting them into human cancer cell lines (and one noncancerous cell line). The phenotypic readouts produced by each compound were classified based on a statistical analysis of cell morphology, staining intensity (staining aids visualization), and the spatial distribution of subcellular structures like nuclei, microtubules, and the Golgi compartments. This analysis could also identify inhibitors of cell components not targeted by known kinase inhibitors.

From the library of screened compounds, Shokat and colleagues identified a molecule (hydroxy-PP) that, though structurally related to a known kinase inhibitor, induced morphological changes distinct from any known kinase inhibitor. What does hydroxy-PP target? An enzyme, called carbonyl reductase 1 (CBR1), that acts on xenobiotics like anticancer drugs and is thought to cause the heart damage associated with daunorubicin chemotherapy.

To better understand how compound and enzyme interact, the authors solved the structure of hydroxy-PP and CBR1 bound together. Knowing their respective structures also suggests ways of enhancing a molecule's effect on a target. In this case, Shokat and colleagues used their structural analysis to increase hydroxy-PP's inhibition of CBR1 in cell culture so they could further explore the enzyme's biological function. These experiments revealed a previously uncharacterized role for CBR1 in programmed cell death.

Given the enzyme's suspected role in chemotherapy-related cardiotoxicity, inhibiting CBR1 activity might enhance the efficacy of chemotherapy treatments by reducing their debilitating side effects—a possibility that future studies can explore. But for now, Shokat and colleagues have demonstrated the power of using high-throughput image-based screening to identify small molecules both for probing cell biology and for identifying promising drug candidates.[Fig pbio-0030165-g001]

**Figure pbio-0030165-g001:**
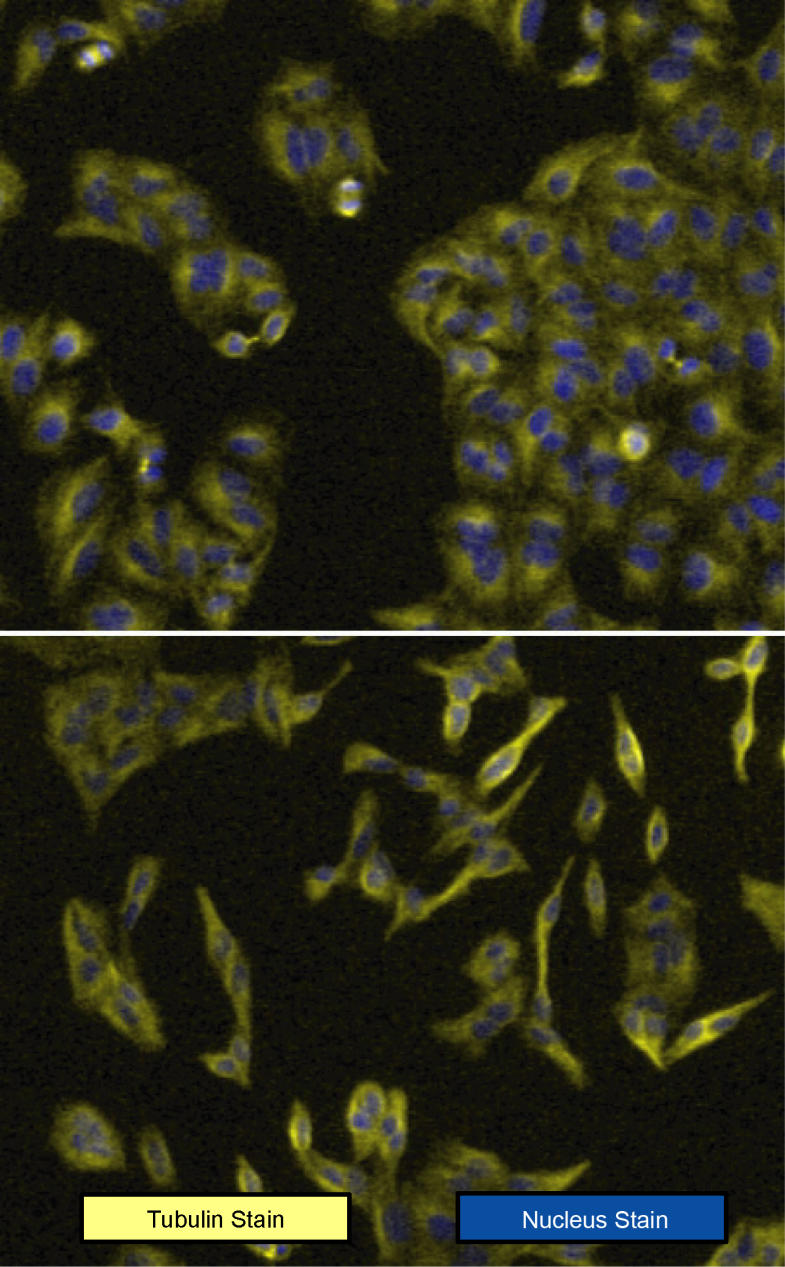
Two closely related compounds produced the morphological differences evident in these lung cancer cells and reveal biological activity that could be important for drug discovery (Photo: Cytokinetics, Inc)

